# Prosocial Behaviors at Work: Key Concepts, Measures, Interventions, Antecedents, and Outcomes

**DOI:** 10.3390/bs14010078

**Published:** 2024-01-22

**Authors:** Rona Hart

**Affiliations:** School of Psychology, University of Sussex, Falmer, Brighton BN1 9RH, UK; rona.hart@sussex.ac.uk; Tel.: +44-7980-709821

## 1. Introduction

At the heart of every thriving organization lies a complex network of personal dynamics, often guided more by human nature than by formal protocols. When examining prosocial behaviors in the workplace, it becomes evident that these behaviors extend beyond company strategies, policies, operational guides, values or norms. It is the innate human drive to connect, contribute, and make a difference that truly shapes an organization’s fabric. From the understated acts of a team member lending a hand to a colleague, to the more significant gestures of altruism that drive teams forward, prosocial behaviors are both a mirror to our motivations and a beacon for organizational harmony.

Prosocial behaviors encompass a wide range of voluntary actions aimed at benefiting others and are recognized as valuable by society [1]. Within a workplace, these behaviors can be performed by an individual, a group, or the organization, and may target anyone from a single person to a group, an organization, a community, or a broader societal objective.

The term prosocial organizational behavior includes a variety of concepts, such as citizenship behavior, civility, respect, care, support, helping, altruism, kindness, collaboration, sharing, cooperation, benevolence, giving, donating, generosity, volunteering, social activism, and heroism to name a few. It also encompasses dispositions such as empathy, sympathy, perspective-taking, humanity and compassion, as well as personality traits such as agreeableness and a prosocial personality. On an organizational level, it involves policies and actions that demonstrate social responsibility, including corporate philanthropy, servant leadership, social enterprise, corporate social responsibility, commitments to diversity, equity, and inclusion, or environmental, social, and corporate governance standards.

In this Special Issue, we embarked on a journey to explore the myriad ways in which prosocial behaviors, however they are conducted, manifest, shape, and transform workplace dynamics.

## 2. An Overview of Published Articles

One of the key critiques of the concept of prosocial behavior is the lack of conceptual clarity. This Special Issue therefore opens with two theoretical papers that help clarify some of the current semantic ambiguities, and offer clearer distinctions between concepts.

In their critical paper, “Untying the Text: Organizational Prosociality and Kindness”, Hart and Hart argued that while the field of organizational prosociality has a long history, there remains a lack of consensus on the definitions of core concepts. The terms ‘prosocial behaviors’ and ‘kindness’ are particularly puzzling, as they are often used interchangeably and have similar definitions, yet their distinctive features remain vague. This leads to conceptual ambiguity within the field, which impedes its progression. The authors undertook a detailed examination of the definitions of prosocial behavior and kindness, clarifying the language used in academic texts, and contextualizing the discussion to the disciplines of psychology and management. They also explored the subtexts that underly these terms. The analysis revealed that both concepts share a focus on dispositions and actions intended to benefit others. Nevertheless, there is a difference in the scope and subjects of these actions. Kindness is an act performed by an individual, targeting another individual or a small group. Prosocial behaviors, on the other hand, can be exhibited by individuals or organizations and may target an individual, a group, or larger entities such as organizations, communities, nations, or even societies.

In another conceptual and critical paper on career calling, Hart and Hart unpacked the intricate features of the career calling concept, drawing on recent reviews which have highlighted ongoing disputes regarding its definition, components, and measurements. A key area of debate centers on the role of prosociality in a calling: whether the motivation behind a calling outlook is primarily self-driven, oriented towards benefiting others, or a combination of both. The paper therefore delves into the pro-self and prosocial aspects of a calling outlook, exploring how these elements manifest in various calling sub-types: classic, neoclassic, and modern callings. The analysis revealed that these calling subtypes differ in their position on a spectrum from pro-self to prosocial motivations: classic callings align with prosocial motivations, modern callings lean towards self-interest, and neoclassic callings occupy a middle ground, blending motivations focused on the self and others. Moreover, the paper revealed that these subtypes are rooted in different value systems: classic callings are driven by the value of self-transcendence, modern callings by self-actualization, and neoclassic callings by a combination of both. The paper concludes by offering definitions for the overarching calling concept as well as for each of its sub-types.

Position papers in the domain of prosocial behaviors often pertain to organizational-level prosocial activity such as corporate social responsibility; diversity, equity, and inclusion; or environmental, social, and corporate governance. In a unique position paper that examines the value of kindness in a healthcare context, Fryburg described the state of the healthcare workplace as inherently stressful, and argued that these heightened and continuous stress levels can impair cognitive abilities, diminishing diagnostic skills, decision-making capabilities, and problem-solving efficiency, and also reduce the likelihood of engaging in helpful behaviors. As stress levels escalate, this can lead to burnout and more severe mental health issues, such as depression and suicidal ideation. One notable effect and contributor to stress is incivility, exhibited by both patients and staff. Such behaviors have been linked to medical errors, which not only have a significant human toll, but also incur substantial economic costs. Consequently, the imperative for fostering kindness in healthcare settings is substantial. Kindness builds positive interpersonal relationships, which can mitigate stress and enhance resilience. Thus, kindness is crucial in the healthcare environment. Strategies to encourage kindness are vital, including leadership demonstrating positive behaviors and discouraging negative ones. The paper also introduces an innovative method to induce kindness involving the use of kindness media.

In “The Prosocial-Culture-Work Nexus: An Integrative Literature Review and Future Research Agenda”, Gibb examined organization culture as a key precursor on the emergence of prosocial behaviors, and found some surprising scholarly gaps. The paper questions what is known about the relationship between prosocial behavior, culture, and work. Research in this area is anticipated to fall into three categories concerning organizational cultures. The first involves studies on specific organizational cultures that embody stated employer value propositions (EVPs). The second category includes etic studies that utilize general constructs of organizational culture. The third consists of emic studies, characterized by the detailed insights typical of ethnography. An integrative literature review focusing on the interplay between prosocial behavior, organizational culture, and work identified 22 studies. The majority of these were etic studies, while the rest were theoretical. Notably, none of these studies seem to apply a clear and consistent definition of organizational culture. The author therefore argued that there is a pressing need to employ and refine constructs of organizational culture.

The following three papers provided a quantitative exploration of the varied outcomes of different types of prosocial dispositions and behaviors.

In “Helping Others Results in Helping Yourself: How Wellbeing Is Shaped by Agreeableness and Perceived Team Cohesion”, Reizer, Harel, and Ben-Shalom reported on a longitudinal study which investigates whether team cohesion serves as a mediator in the relationship between the prosocial personality trait of agreeableness and mental wellbeing. It also assessed whether the level of support provided by a leader affects perceived team cohesion. The participants were 648 male military personnel from six units. The research spanned two time points, T1 and T2, during the soldiers’ training period. The findings revealed that agreeableness and team cohesion at T1 predicted enhanced wellbeing at T2, occurring two months later. The results also supported the moderated mediation hypothesis, showing that the indirect relationship between agreeableness and wellbeing through team cohesion was stronger in instances of greater leader support compared to when leader support was low.

Following a similar line of research which aimed to assess whether prosocial behaviors predict work wellbeing, Santos, Lousa, Sa, and Cordeiro explored organizational citizenship behavior (OCB), which includes two sub-types: individual-directed organizational citizenship behaviors (OCBI) and organizational-directed organizational citizenship behaviors (OCBO). The research also explored how different leadership styles, specifically those centered on people or tasks, moderate the relationship between OCB and work wellbeing. The participants were 200 employees from various organizations. The findings indicated that OCB has a positive and significant influence on work wellbeing, with individual-directed OCBI having a more pronounced impact than organizational-directed OCBO. Surprisingly, the direct link between leadership styles and wellbeing was not statistically significant. Nevertheless, the study did find that leadership styles moderated the effect of OCB on wellbeing at work, with one exception: this moderating effect was not observed when employees engaged in OCBO under a people-focused leadership style.

In another study which investigated the outcomes of OCB in part-time and temporary working university students, Johansson and Hart examined whether OCB predicts various outcomes, including job stress, work-university conflict, work–leisure conflict, intentions to leave a job, psychological wellbeing, and job satisfaction. The study gathered data from 122 employed university students. The results revealed that OCB was positively associated with work–university and work–leisure conflicts. However, there was no significant correlation between OCB and wellbeing, stress, job satisfaction, or intentions to quit. The regression analyses indicated that OCB is a positive predictor of job satisfaction when entered alongside work–university conflict, job stress, and intentions to quit. Additionally, OCB was found to predict job stress when entered with job satisfaction, but it did not predict wellbeing. These results highlight the negative consequences of OCB as well as diverging from the existing body of research on full-time employees.

The following three quantitative papers took the opposite approach and explored the predictors of various types of prosocial behaviors.

In “Positive Impact, Creativity, and Innovative Behavior at Work: The Mediating Role of Basic Needs Satisfaction”, Papachristopoulos, Dubord, Jauvin, Forest, and Coulombe drew on self-determination theory to propose that the satisfaction of basic psychological needs and prosocial action in the form of benevolence could be a key intermediary linking an employee’s perception of their prosocial impact to their innovative and creative work behaviors. The study, involving 528 participants, found that both perceived prosocial impact and prosocial motivation are positively linked to innovative work behavior and creativity. Additionally, the satisfaction of the needs of autonomy and competence was identified as a mediator between perceived social impact and the work outcomes. Interestingly, the study also discovered that prosocial motivation moderated the relationship between benevolence and innovation.

Taking a similar line of research, Su and Hahn explored several factors that might predict OCB in construction workers, including psychological capital (PsyCap), prosocial motivation, and perceived corporate social responsibility (CSR) as predictors. Hypotheses were tested using 336 questionnaire responses from 56 teams. The findings revealed that PsyCap significantly predicts employees’ OCB, and a positive correlation was found between PsyCap and employees’ prosocial motivation, with the latter partially mediating the PsyCap-OCB relationship. CSR was found to influence the PsyCap-prosocial motivation link and significantly moderate the connection between prosocial motivation and OCB.

In “Determinants of Preschool Teachers’ Knowledge-Sharing Behavior from a Thinking Style Perspective”, Cheng, Fu, and Cao explored the factors that may influence preschool teachers’ knowledge-sharing behavior, including thinking style, awareness of consequences, ascription of responsibility, and personal norms. An analysis of the data collected from 297 teachers revealed that teachers with an executive thinking style displayed a notable awareness of consequences, while those with a legislative thinking style showed both awareness of consequences and ascription of responsibility. The study found that awareness of consequences significantly boosted ascription of responsibility, which, along with awareness of consequences, positively affected personal norms. These personal norms, in turn, positively influence knowledge-sharing behavior.

In “The Divergent Effects of the Public’s Sense of Power on Donation Intention”, Yuan, Li, and Ju explored whether one’s sense of power, situational regulatory focus, perceived ethical climate and demographic variables predict financial donation intentions which are either improvement-based or avoidance-based. The study was conducted through a three-wave time-lagged survey involving 1200 participants. The findings revealed that situational prevention focus acts as a mediator in the relationship between a sense of power and avoidance-based donation intention. Similarly, situational promotion focus mediates the relationship between a sense of power and improvement-based donation intention.

Lastly, in the only qualitative study included in this volume, Barton and Hart explored the experience of self-transcendence in social activists. Employing constructivist grounded theory, this study conducted in-depth semi-structured interviews with eight individuals who self-identify as self-transcendent social activists. These participants are characterized by their initiation of voluntary, non-profit community actions. The analysis focused on examining the personal experiences of self-transcendence among participants and how their self-transcendent nature influenced their decisions to engage in social activism. The results of this study offer a definition of ‘self-transcendent social activism’ and introduced a theoretical model that outlines the progression of participants’ activism through several developmental stages: trigger, activate, maintain, and sustain. This progression led to impacts experienced on three levels: individual, community, and global.

## 3. Conclusions

In concluding this Special Issue, it is worth highlighting the diverse types of research and topics included, ranging from conceptual papers to empirical, covering individual and organizational prosocial behaviors and actions, and looking both at the consequences of prosocial behaviors as well as what predicts prosocial behaviors. Collectively, these papers contribute to our understanding of prosocial behaviors but also offer practical insights for fostering such behaviors in various workplace settings. As we reflect on these contributions, it is our hope that this Special Issue will serve as a valuable resource for researchers, practitioners, and policymakers alike, inspiring the continued exploration and application of prosocial behaviors in the pursuit of more cooperative, supportive, and thriving work environments.
